# Recent progress and perspectives of space electric propulsion systems based on smart nanomaterials

**DOI:** 10.1038/s41467-017-02269-7

**Published:** 2018-02-28

**Authors:** I. Levchenko, S. Xu, G. Teel, D. Mariotti, M. L. R. Walker, M. Keidar

**Affiliations:** 1 Plasma Sources and Applications Centre, National Institute of Education, Nanyang Technological University, 1 Nanyang Walk, Singapore, 637616 Singapore; 2 School of Chemistry, Physics, and Mechanical Engineering, Queensland University of Technology (QUT), Brisbane, QLD 4000 Australia; 3 Department of Mechanical and Aerospace Engineering, The George Washington University, Washington, DC 20052 USA; 4 Nanotechnology and Integrated Bio-Engineering Centre (NIBEC), Ulster University, Newtownabbey, BT37 0QB UK; 5 School of Aerospace Engineering, Georgia Institute of Technology, Atlanta, GA 30332-0150 USA

## Abstract

Drastic miniaturization of electronics and ingression of next-generation nanomaterials into space technology have provoked a renaissance in interplanetary flights and near-Earth space exploration using small unmanned satellites and systems. As the next stage, the NASA’s 2015 Nanotechnology Roadmap initiative called for new design paradigms that integrate nanotechnology and conceptually new materials to build advanced, deep-space-capable, adaptive spacecraft. This review examines the cutting edge and discusses the opportunities for integration of nanomaterials into the most advanced types of electric propulsion devices that take advantage of their unique features and boost their efficiency and service life. Finally, we propose a concept of an adaptive thruster.

## Abstract (web summary)

Miniaturized spacecraft built from advanced nanomaterials are poised for unmanned space exploration. In this review, the authors examine the integration of nanotechnology in electric propulsion systems and propose the concept of self-healing and adaptive thrusters.

## Introduction

Major progress in robotics and microelectronics, as well as significant advances in nanoelectronics, make it possible to efficiently explore both near Earth and deep space with small spacecraft^1^. These spacecraft and ultra-small satellites, sometimes referred to as Cubesats, are poised to permanently transform the global economy and mankind’s approach to space exploration. In particular, small spacecraft have dramatically lowered the initial capital cost of deploying on-orbit capability. Historically, only large companies and government organizations could meet the cost (>$100M USD) of manufacturing a functional space asset, purchasing the launch, and acquiring insurance. Small satellites, enabled by the miniaturization of electronics and related systems, have brought the entry capital barrier down to as low as $1M USD. This has allowed new players, such as universities and small companies, to acquire custom on-orbit assets. Launch providers have added fuel to this fire with the introduction of numerous, low-cost secondary payload rides.

Small satellites and Cubesats, as well as their related capability have touched all aspects of human activity. Private investors have embraced the use of the small surveillance platforms to monitor natural resources and commerce activity. These companies include Terra Bella (formerly Google Skybox) and Planet. In response to the growing demand for small satellite technology, a variety of small businesses has appeared to support the component needs of satellites suppliers. Cubesats exist already on almost every University campus and span the spectrum from large research institutions such as the Georgia Institute of Technology to small community colleges, e.g., Medger Evers College. A plethora of organizations associated with defense and surveillance actively use small satellite assets to conduct their intelligence missions. The Air Force continues to build the skilled workforce through sponsorship of University Nanosat Program. Civil space organizations have focused multiple programs and solicitations on Smallsat technology development as well as on leveraging small satellite platforms to perform unique science missions. Multiple NASA centers develop and deploy small satellites and Cubesats from the International Space Station. Box 1 shows the growth in the number of publications related to satellites and relevant systems, reflecting a strong increase in interest in this field.

While small satellites have enjoyed initial success, the civil sector requires more sophisticated spacecraft to perform remote Earth sensing, precision agriculture, monitoring erosion and sea pollution, precision weather prediction, navigation, global satellite-based communication systems, and many other tasks that could provide a considerable boost to the efficacy of these areas of human activity^2^. Moreover, smart robotic orbiters and landers could efficiently explore other planets including Venus and Mars. Apparently, the next steps in the use of small satellites and Cubesats to perform civil space missions could be divided into two categories, (i) exploring our solar system and beyond, and (ii) leveraging the near-Earth space. Missions in our solar system include further exploration of the Moon^3^, exploration of Mars^4,5^, and sending long-life probes to planets, comets, asteroids^6^, and deep space^7^. In parallel, intense use of near-Earth space will continue to benefit the entire mankind**—**from using small space assets for advanced communication, global internet access, precise weather prediction, asteroid tracking, global positioning systems, to near-Earth space exploration (e.g., registration of radiation and corpuscular fluxes, and their effect on communication systems and weather)^8,9^. These missions demand very sophisticated, autonomous equipment that reliably operates within the hostile space environment**—**very low and high temperatures, extreme radiation, impact by high-speed dust particles, and other severe conditions. In this review, we provide a brief literature survey on the unique features of nanostructured materials in the context of their application in electric propulsion (EP) systems and thrusters, with a strong focus on Hall-effect and gridded ion thrusters. In particular, the application of vertically aligned nanotube patterns, nanotube- and graphene-based nanomaterials, as well as complex metamaterials involving nanoscaled structures and related physical effects are discussed. Moreover, the concepts of adaptive and self-healing thrusters are proposed, presenting systems capable of self-adjusting the operation mode. Development of a new generation of miniaturized EP platforms is expected, based on the ingression of nanomaterials into space technology.

### Electric propulsion systems as the first choice

Wide application of nanoapproach in space technology could be a possible way to realize smart, nanoscale spacecraft. According to the NASA’s 2015 Nanotechnology Roadmap Initiative, nanomaterials and related techniques should constitute a base for the new generation of spacecraft^10^. Evidently, EP systems are the primary candidates for driving the development of these novel spacecrafts—see Boxes 2 and 3 to learn more about design and impressive benefits of the EP platforms and the two most advanced types of EP devices, namely Hall-type and gridded ion thrusters.

Why is EP unique? Powerful liquid and solid propellant rocket engines are commonly used during the first stages of space flight. Despite relatively low specific impulse^11^ (i.e., propellant exhaust velocity), they feature very high thrust-to-weight ratios. Thus, they are most suitable for launch applications and reaching orbital velocity. In contrast, most long-duration missions imply low thrust but require high specific impulse to efficiently control the orientation and location of the spacecraft. Unfortunately, small chemical rocket engines deliver very low specific impulse at low thrust levels. In contrast, the EP that uses electrical energy to accelerate ionized propellant and delivers very high specific impulse is the leading propulsion solution for long-duration missions^12^. EP devices employ electrostatic or electromagnetic forces to accelerate the propellant and thus do not have physical limitations on their exhaust velocity.

In terms of specific impulse (which shows how efficiently the propellant is used), this means that EP can develop thrust at a very low propellant mass flow rate, i.e., the mass of propellant required for the entire mission could be small. Such a system is the best candidate for long-duration spacecraft control^13^. Thrusters employing electrostatic methods of acceleration (specifically Hall- and gridded ion-type devices) are superior to other electric types due to low secondary losses of energy (e.g., for ionization of propellant and heating), and they are the most promising platforms for deep space and interplanetary flights. Other devices such as pulsed plasma thrusters^14^, magnetoplasmadynamic thrusters, and novel perspective approaches, e.g., the variable specific impulse magnetoplasma rocket (VASIMIR^15^, are also suitable for specific space tasks). It is important to remember that because EP devices use electrical power to accelerate the propellant, their operation is power limited.

**Fig. 1:**
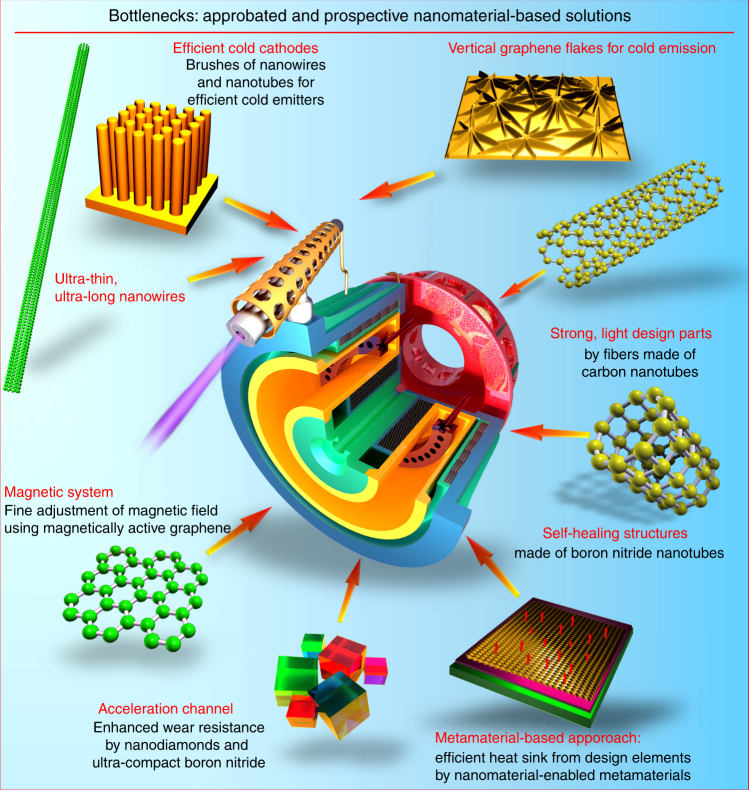
Bottlenecks and nanomaterial-based solutions for electric thruster. Reduction of the ion flux-related wear of an acceleration channel material and enhancement of discharge efficiency by using, e.g., nanodiamond coatings and ultra-compact boron nitride; enhancement of cathode efficiency by using cold emission insets made of dense brushes of long, ultra-thing nanotubes, nanowires, and vertically oriented graphene flakes; optimization of the magnetic circuit by the use of magnet-active graphenes; application of light, durable materials made of carbon nanotube yarns for the propulsion system parts are among the major challenges

The general aim of this review article is to demonstrate encouraging examples and possible approaches for boosting the performance and lifespan of spacecraft EP systems by the wide-and-wise application of nanoscaled materials and techniques in the most challenging, critical areas (see Fig. 1 for the most determined “bottlenecks” to be possibly resolved with the use of the next-generation nanomaterials and metamaterial systems). Eventually, we try to set the major directions and approaches for self-healing thruster platforms suitable for the ultra-long deep space missions.

### Box 1: Publications on thrusters and satellites

**Growth in the number of publications on electric propulsion and satellites**. The results of the search for “plasma thruster” (**a**) and “satellites” (**b**) keywords in publications from American Institute of Physics (AIP), Institute of Electrical and Electronics Engineers (IEEE), Institute of Physics (IOP), Elsevier, and the American Institute of Aeronautics and Astronautics (AIAA). Considerable increase in the numbers of accepted papers indicates strong and growing interest in the field. (**c**) An example of publication distribution across several popular academic journals.
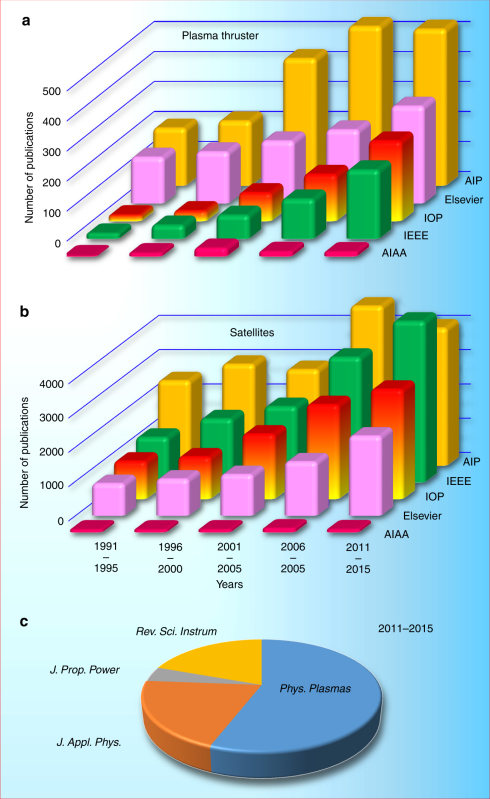


### Box 2: plasma thruster as a unique propulsor

**Electric propulsion—why is it the first choice?**

While successfully used to launch and gain orbital velocity, thrusters based on heat engines (liquid and solid propellant rocket engines) feature essentially low mass efficiency due to the thermodynamic limitations. One more drawback of the heat engines is their extremely low efficiency at small thrust, the latter being a quality essential for continuously operating systems to ensure permanent adaptive control of the spacecraft orientation and orbit keeping.

The electric thrusters do not pose limits on the propellant exhaust velocity *V*_ex_ (in contrast to the thermal engines where *V*_ex_ is limited by possible enthalpy, and specifically, the most efficient F + H_2_ fuel is limited to *V*_ex_≈6000 m/s), and the propellant mass consumption may be very low according to the relation:

1 $$F = \dot mV_{{\mathrm{ex}}},$$

where $\dot m$ stands for mass consumption, kg/s, and *F* is the thrust, *N*. The power *ɛ* required to accelerate the exhausted mass (i.e., the minimum power required to create the thrust, assuming 100% fuel efficiency) is

2 $$\varepsilon = \frac{{\dot mV_{{\mathrm{ex}}}^2}}{2},$$

where *V*_ex_ is the exhaust velocity, m/s. Combining Eq. 1 and Eq. 2, we obtain an important relation for the thrust via power applied to mass acceleration, and exhaust velocity:

3 $$F = \frac{{2\varepsilon }}{{V_{ex}}},$$

which shows that mass flux, $\dot m$ actually is not a physical limitation for thrust, and thus virtually any thrust may be obtained for the indefinitely small $\dot m$, provided that enough power is spent. We recall here that in any type of thermal engine where the energy is stored in the chemically active fuel, physical limitations exist, making it impossible to increase the power applied to the exhausted mass.

Image on the right illustrates a composition of the typical EP system consisting of several major subsystems including a gas supply system, an energy supply system, a control system, and the thruster itself.

See Box 3 for a short description of the two major types of EP thrusters.
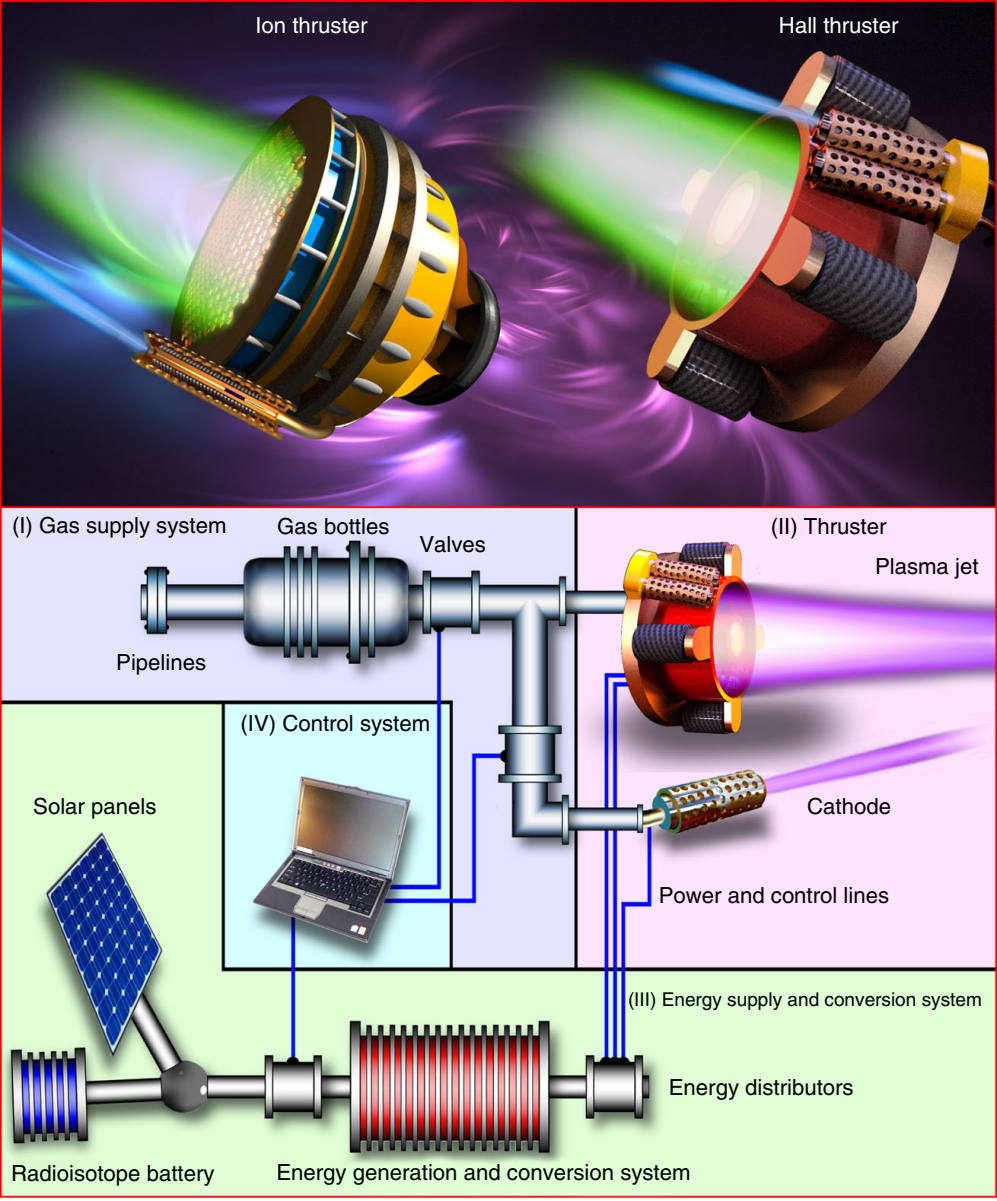


### Box 3: structure and operation of Hall and ion thrusters



**Hall thruster—what are the main parts?** A Hall thruster incorporates several external magnetic coils or one large coil embracing the whole thruster, and an optional internal magnetic coil. An incandescent cathode is usually installed outside the accelerating channel, and anode is installed in the channel. Ceramic walls of the accelerating channel may be replaced with a metallic insert; such a variation is called thruster with anode layer (TAL).

**How does it work?** Propellant gas (typically Xe) is supplied to the accelerating channel through holes in the anode (see figure to the left). Electric potential is applied to the external cathode relative to the anode. A closed Hall current is generated in the channel due to crossed electric and magnetic fields. While ions are accelerated by the static electric field, the magnetized electrons participating in the circular Hall current diffuse toward the anode.

Hall thrusters are essentially electrostatic ion accelerators, but their thrust can be expressed in terms of the interaction of circular electron current with the external magnetic field. Detailed description of the ion thruster and the basic principles of its operation can be found elsewhere (see numerous references in the text).

**Ion thruster—what are the main parts?** An ion thruster consists of a cylindrical body with the discharge unit comprising an incandescent hollow cathode, an anode, and a magnetic coil; several focusing magnetic coils installed over the body, and acceleration mesh unit comprising (typically) an internal extracting mesh and an external acceleration mesh. The external cathode is installed on the body outside of the thruster.

**How does it work?** Propellant gas (typically Xe) is supplied to the hollow cathode unit where it is ionized in the magnetized discharge. The ion flux is extracted from the plasma by the internal mesh, and finally accelerated by the second (acceleration) mesh. High-velocity ion flux is ejected off the thruster, and an electron flux from the external cathode compensates for the electric charge of the ion flux. As a result, neutral plasma flows off the thruster, creating no charge on the spacecraft. Detailed description of the ion thruster structure and its principles of operation can be found elsewhere (see references in the text).

It should be noted that in both Hall and ion thrusters, the plasma is maintained due to the electron-neutral collisions. Apparently, this is not the only available method, and other ionization routes such as radiofrequency and microwaves could be used in thrusters^143^.

**Thrusters in space and on Earth**: left photograph (**a**), four small EP thrusters installed on a Cubesat ready for the orbital flight^144^. Courtesy of Dr. Jin Kang. Central photo (**b**), a STENTOR EP module made of one SPT-100 (Hall-type thruster of 100 mm size) and one PPS-1350 (designed by SNECMA Moteurs for geostationary telecommunication satellites) devices mounted on an ALCATEL gimbals mechanism. Circular accelerating channels and twinned cathodes are clearly visible. Reproduced from ref. ^8^. (Copyright 2005 ERPS). Right photo (**c**), large EP facility test chamber at the Space and Propulsion Center, Singapore.

See Box 4 for a short description of the pioneering experiments on self-healing in EP thrusters.

### Hall-type and ion thrusters as the two main candidates

Hall-type and gridded ion thrusters are among the most advanced, mature EP technologies with space-proven, relatively long flight heritage; some prominent EP-driven missions are described in more detail below. Specifically, a Hall-type thruster is a device that uses the closed electron drift, so-called Hall current, as the principal physical effect to drive the main processes in the discharge chamber, namely propellant gas ionization and ion flux acceleration via a static electric field^16^. Compared to other types of plasma thrusters^17^, the devices based on the closed electron drift possess very high efficiency due to low ionization losses and the absence of actively heated parts in the discharge zone.

The Hall thrusters are used for creating small (up to ~250 mN) thrust levels^18,19^, though no principal physical limitations are known to compromise the operation of Hall thrusters at power levels higher than 100 kW^20^ (actually, efficiency increases at higher power levels). These devices have also been successfully scaled down to powers lower than 100 W at μN-thrust levels^21^. Besides, Hall thrusters can produce a very large number (>10^4^) of thrust pulses without undergoing maintenance and part replacement, with a very high (up to 40–50 × 10^3^ m/s) exhaust velocity^22,23^.

In the gridded ion plasma thruster, the electric potential is applied to the acceleration mesh, and thus the ion flux is accelerated and expelled from the thruster through the mesh. Both types of thrusters are very efficient devices for plasma production and acceleration. One significant difference between them is the current density limitation applied to the ion thruster due to the space charge limitation; this results in a larger ion thruster diameter compared to the Hall thruster. More details and illustrations can be found in Boxes.

Are they flying? The EP systems are already performing missions in space, thus accumulating invaluable experience and paving the way for the future. In fact, these systems possess a long history that started in 1906^24^, or more realistically, in 1950^25^. The first ever EP flight tests using ablative pulsed plasma thrusters took place in 1964^26^. The first flight demonstration of the Hall thruster occurred in 1971, and the subsequent flights have proven the high reliability and efficiency of this type of the device^27^. Launched in 1998, the Deep Space 1 probe was accelerated by >4000 m/s using 2-kW ion thrusters^28^, which spent about 70 kg of xenon exhausted at a speed of 4 × 10^4^ m/s for near 2 years^29^. A similar system was installed on the gravity-ocean circulation explorer GOCE satellite in 2009^30^. Currently, the SSL SPT-100 Hall thruster subsystem is flying on 17 geostationary communication spacecraft, with an accumulated total flight time of 40,000+ h^9^. ADD Aerojet Rocketdyne on LM AEHF satellites Eutelsat has demonstrated the EP system capabilities on two platforms (EUTELSAT 16C and KA-SAT), and launched the full-electric platform EUTELSAT 115 West B in March 2015, for which EP was used for complete electric orbit raising. Two more platforms (EUTELSAT 117 West B and EUTELSAT 172 B) will be also launched^31^. The PROITERES-3 nanosatellite equipped with 30-W Hall thruster is planned for a one-way trip to the moon from low orbit^21^. However, the long-expected era of electrically driven spacecraft has yet to be realized, where the main obstacle lies in the relatively high consumption of electric energy by the thrusters, requiring significant enhancement in the thruster efficiency^32^.

Recently, NASA has designed and performed the DAWN mission to investigate the two main-belt asteroids, Vesta and Ceres, which are among the most massive small planets. The DAWN mission was actually the ninth project of the Discovery Program. Except for the gravitational maneuver in the Mars’ gravitational field, this mission was powered exclusively by an EP system. The entire post-launch velocity gain Δ*V* required for the heliocentric transfer to Vesta, then retardation to capture orbit at Vesta, additional acceleration for changing orbits at Vesta, final escape from orbit Vesta, then transfer to Ceres by heliocentric orbit, retardation to capture orbit at Ceres, and the acceleration to change orbits at Ceres was provided by the ion thrusters. The EP platform in this mission has ensured the total velocity gain of ~11 km/s, and ~400 kg of xenon was spent during the entire mission^28,33^.

Another important option for the application of EP systems is the control of low mass (up to 100 kg) and miniature (Cubesats, up to ≈1 kg) satellites that have a base form factor of a cube with the typical size of 0.1 m. An internationally adopted standard in this field is the “Cubesat” by California Polytechnic State University (CalPoly)^34^. Miniaturized but fully functional^35^, highly efficient modern electronics ensure a wide spectrum of capabilities even on such a small scale, thus enabling a great resource economy due to low-cost launching using, e.g., small airborne systems like the Orbital ATK Pegasus^36^. The propulsion systems used on Cubesats usually provide a thrust of up to 1 mN, with the consumed power of up to 100 W and the unit mass of several kg^37,38^. Recently, a so-called “satellite-on-a-chip” or “ChipSat” concept was proposed with the aim to explore near-Earth space using the ultra-miniaturized spacecraft in the form of monolithic semiconductor integrated circuits, with a weight not exceeding 0.1 g (“smart dust”)^39^, as well as picosatellites (0.1–1 kg), and femtosatellites (up to 0.1 kg)^40^. A large number of the ultra-miniaturized satellites (smart dust nodes) forming an actively interacting distributed network could provide a highly efficient solution for collecting, converting, and transmitting large amounts of information^41^. Gas and thermal thrusters are not applicable in this case, and an EP system may be the only possible option^42^.

Are they efficient? Yes, even at their present stage of development they are quite efficient. The efficiency of contemporary EP systems reaches 10–35% for the most common power range of 100–300 W^43^, downscaling (depending on the design) to 6–15% in the power range of 50–100 W^44^. Larger thrusters demonstrate higher efficiency, reaching 40–60%^45^ at a power range of 1–3 kW and even 65% for higher powers^46^—these are very large numbers for any heat engine. Nevertheless, significant advances in power efficiency and controllability, as well as service life are needed to make the EP systems the cornerstone of space propulsion capable of highly efficient, robust operation under severe space conditions for years.

As for now, significant efforts have been made to enhance the performance, service life, and reliability of the Hall thruster by means of sophisticated designs. Among others, the two-stage Hall thruster was designed and tested^47^. To ensure high performance at very high power levels reaching 100 kW, a multiple nested thruster designs have been demonstrated^20^. Other design solutions such as cylindrical and annular Hall thrusters were proposed for the miniaturized devices^44^. More details on the methodology and problems related to scaling down EP devices could be found in the relevant publications^48,49^. Nevertheless, further improvements are still required, where the major advancements should definitely come from the material field.

How can we boost their performance and service life? The performance of contemporary EP devices can be boosted by the application of ultra-novel nanoscaled materials and systems to significantly enhance work capabilities and efficiency of all subsystems of the EP system and the most important units of thrusters, including ion and Hall thrusters specifically^50^. Significant progress has already been achieved in the optimization of the design, geometry, and magnetic field configuration of the thrusters; however, further progress is vitally required to launch plasma thrusters into orbit of a commercially efficient life, and we propose and review here the use of surface-bound nanostructures and nano-engineered surfaces as a next-generation approach to the development of EP systems. Evidently, all the main challenges, namely efficiency, lifetime, and process control of the thruster can be significantly enhanced by the use of nanoscaled materials and techniques.

There are other types of plasma thrusters potentially suitable for Cubesats and even smaller satellites. While Hall-type and gridded ion thrusters enjoy a relatively established reputation among small orbit- and interplanet-tested systems, other types of EP devices are just at the start of their road to the stars. The rise of Cubesats and ultra-small (Picosats**—<**1 kg mass) satellites spurred global demand for micro-N propulsors, and the first launches have already proven their suitability and efficiency for the specific Cubesat tasks**—**precise positioning, orientation, and orbit keeping with µ-N pulses. These thrusters are designed to operate at ultra-small thrust levels, providing a superior level of control over satellite positioning and orientation; however, the material issues still play an important role in their advancement and integration into existing and emerging satellite systems^51^.

### Nanomaterials in thrusters

Let us now examine how to boost the properties and characteristics of the thruster itself, which is the primary and critical sub-subsystem of the entire EP platform.

**Fig. 2:**
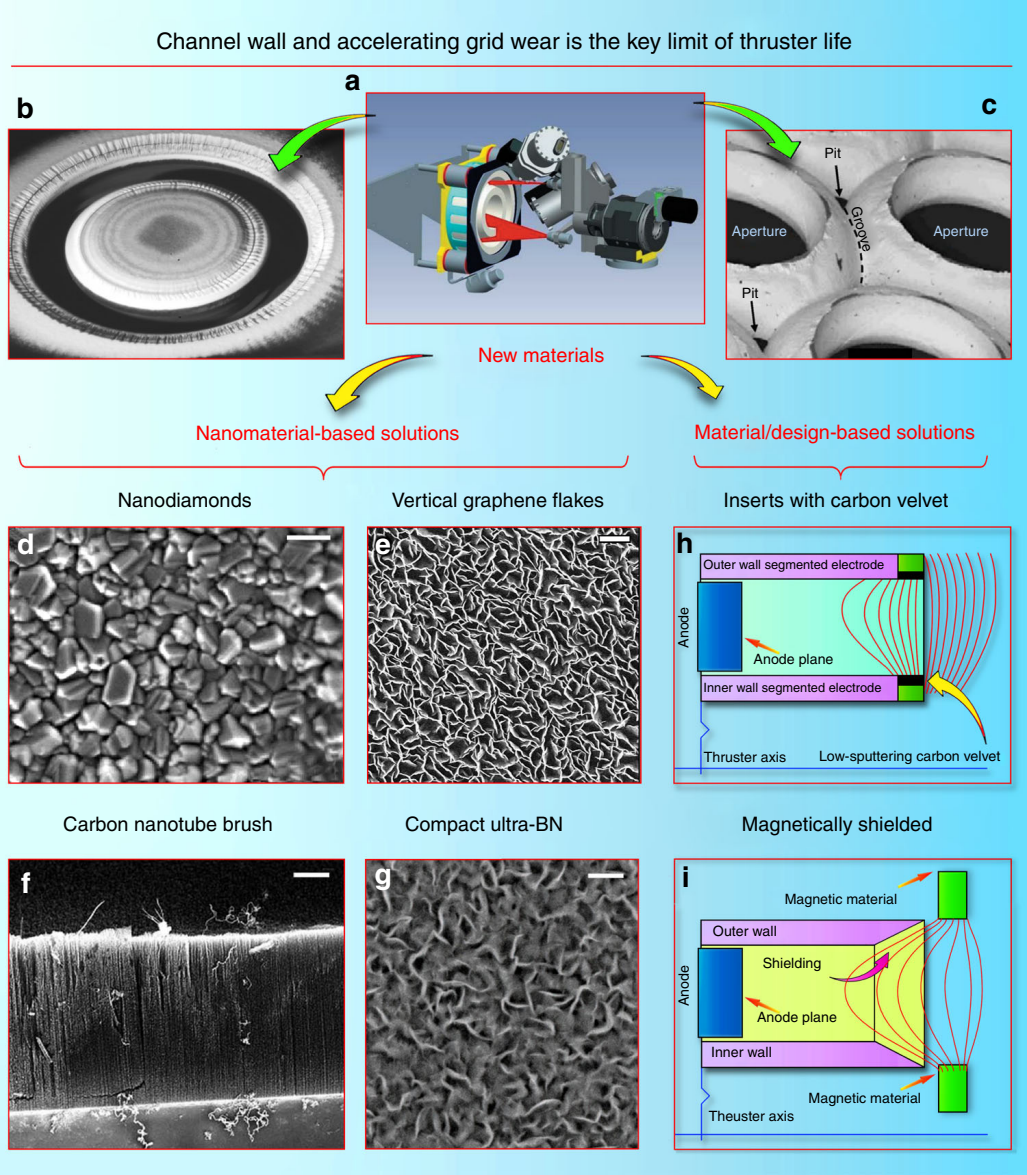
Nanomaterials to reduce erosion of accelerating grids and channels. Erosion measurement and analysis is not a trivial problem; **a** illustrates a Telemicroscopy Erosion Measurement System for 5-kW-class Hall effect thruster. Reproduced from ref. ^125^. (Copyright 2015 ERPS). **b** Erosion of the Hall thruster acceleration channel. Reproduced from ref. ^126^. (Courtesy Aerojet Rocketdyne, Inc., Copyright Aerojet Rocketdyne, Inc. 2010). **c** Erosion of the ion thruster acceleration grid. Reproduced from ref. ^127^. (Copyright 2008, IEEE). Wear damage of several millimeters results in a significant drop in thruster efficiency. Nanomaterials-based solutions: (**d**) surface morphology of nanodiamond films with nanocrystalls of 100 nm and a film layer of 600 nm were fabricated at 870 °C. Scale bar, 0.1 µm. Reproduced from ref. ^128^. (Copyright AIP 2012). Nanodiamonds were successfully tested in the thruster channel^57^. **e** Vertical carbon nanoflakes grown on an alumina membrane could potentially help to reduce wear. Scale bar, 1 µm. Reproduced from ref. ^129^. (Copyright 2014, Elsevier). **f** SEM image of the typical carbon nanotube brushes. Scale bar, 5 µm. Reproduced from ref. ^130^. (Copyright RSC, 2015). **g** A compact magnetron-deposited low-sputtering boron nitride coating (synthesized in authors’ laboratory). Scale bar, 400 nm. This material has demonstrated the total wear rate of several nm × h^−1^, which corresponds to only 20 µm per 10,000 h. To compare, the typical erosion numbers are: Al_2_O_3_—0.3 µm/h, diamond—0.7 µm/h, SiC—0.8 µm/h, and BN—1.1 µm/h^131^, with 5000 h service time as a good benchmark for today’s thrusters, such as a Snecma Safran Group device that completed a successful mission to the Moon^132^. Material/design-based solutions: (**h**) schematics of a Hall-type thruster channel with insets covered with the low-sputtering carbon velvets^67, 133^. **i** The magnetically shielded channel solution requires precise tuning of the magnetic field, but significantly reduces the channel wall sputtering yield^74, 134^

Acceleration channel**—**what is the main challenge? One of the major problems limiting the application of Hall thrusters in space is the erosion of the ceramic wall caused by the impact of the accelerated ion flux (Fig. 2)^52^. With the ion velocity reaching 10-–20 × 10^3^ m/s, the wall wear is non negligible, significantly limiting the thruster life^53^. Currently, used boron nitride (BN) ceramics ensure remarkable operating characteristics but unsatisfactory service life;^54^ many researchers are currently dealing with this problem^55^. Design efforts have aimed to reduce the wall wear, for example, by using a cusp-type magnetic field that leads to a simplified (no internal ceramic wall) channel geometry, thus lowering integral wear; however, the wall material is at the heart of this problem. Table 1 provides a comparison of several most popular materials tested in the thruster conditions.

**Table 1:** Currently used and proposed materials for thrusters

Material	Tested	Ref.
Boron nitride, BN	Tested, in use, high efficiency, low erosion (0.02 mm^3^/C at 300 V)	^131, 139, 140^
Borosil, BN + SiO_2_	Tested, in use, high efficiency, low wear	^131, 141^
Alumina, Al_2_O_3_	Tested, medium efficiency, low erosion (0.01 mm^3^/C at 300 V)	^139, 141^
Silicon carbide, SiC	Tested, medium efficiency, medium erosion	^131^
Graphite	Tested, low efficiency, high erosion	^65^
Nanocrystalline diamond	Tested, high efficiency, low erosion	^131, 142^
CVD diamond	Tested for wear, lower erosion	^63^
Ultra-BN, uBN	Tested, high efficiency, very low wear	New result

Ultrananocrystalline diamond may be a promising approach to prolong service life. The first possible attempt to reduce wall erosion is to use thin sputtered films of a nanocrystalline wear-resistant material^56^ such as diamond. Polycrystalline chemical vapor-deposited diamond films were tested on Hall thruster walls (on wear-affected areas), and have demonstrated significantly better wear resistance without notable degradation of the thruster performance and thrust^57^. Evidently, the choice of the wall materials significantly affects the plasma discharge via changes in the secondary electron emission, and more efforts should be applied to study all aspects of this process; nevertheless, these encouraging results attract special attention to the crystalline nanodiamond, which can be a very promising candidate to address a significant increase in wear-limited life. Indeed, it was demonstrated that the as-grown ultrananocrystalline diamond films have wear coefficients roughly two orders of magnitude lower than those of microcrystalline diamond films of comparable thickness^58^. Other tests^59^ have demonstrated absent or negligible wear of the ultrananocrystalline diamond films under conditions when other wear-resistant parts suffered significant wear rates^60^. In mechanical tests, coatings of ultrananocrystalline diamond have demonstrated an order of magnitude longer lifetime^61^. Such inspiring results obtained for the ultrananocrystalline diamond suggest the need for extensive tests of ultrananocrystalline diamond films for the wear reduction of the Hall channel walls. It should be noted that the morphology and surface condition of the ultrananocrystalline diamond strongly affects its tribological behavior^62^, while the properties of the Hall thruster itself critically depend on the wall conditions. Therefore, careful design and both experimental and analytical examinations will be needed to implement this sophisticated nanomaterial in the EP technique, and thorough testing of the actual effect of energetic ion flux directly in the thruster channel is required. Nevertheless, breakthrough results may be expected and the thruster efficiency may be significantly boosted.

Even more sophisticated nanomaterial with the promising wear-resistance properties was also demonstrated. Specifically, the hot-filament chemical vapor deposition was used to synthesize nanocrystalline diamond-coated silicon nitride ceramics^63^. This approach may be useful for enhancing the thruster wall wear resistance and simultaneously adjusting the acceleration process parameters, such as wall conductivity, roughness, secondary emission coefficient, and others that directly influence the discharge^64^.

Carbon can be also used for channel enhancement. One more promising approach to reduce channel wall erosion is the use of carbon-based nanostructured materials and surface structures. Carbon exhibits a very low sputtering rate under the action of ion flux, as compared with the commonly used BN ceramics^65^. Along with this, the secondary electron emission yield of carbon is also lower than that of BN, and this is also a useful feature that can be a factor in Hall thruster operation^66^. We should stress that BN demonstrates one of the best performances as a wall material, so at the first stage, the aim should be the design of the material that can at least preserve the efficiency of BN state-of-the-art thrusters. On the other hand, increase of wear resistance by the factor of 2 or 3 due to the wear-resistant carbon films^67^ is attractive for enhancing the Hall thruster lifetime.

Carbon nanotubes are also a promising technique to enhance channel wear resistance. It is known that the graphene and graphene-based nanostructures, such as carbon nanotubes, are very strong (strongest known in nature) materials^68^. The carbon nanotubes were also tested for resistance against ion flux erosion. Specifically, the multiwall carbon nanotubes were tested as the protective coating against plasma erosion in advanced space propulsion systems. The polycrystalline diamond film was compared with multiwall nanotubes, amorphous carbon, and BN films^69^. Two types of nanotubes were investigated, including vertically aligned nanotubes and those horizontally laid on the substrate surfaces. Only diamond films and vertically aligned nanotubes survived erosion by 250 eV krypton ions of a flight-quality Hall thrusters^69^.

It should be stressed that the use of carbon nanotubes in the thruster is presently at the stage of an advanced concept that requires strong efforts to check feasibility. Indeed, many properties specific to carbon nanotubes, such as a decrease of the secondary electron emission in vertically aligned structures and electrical conductivity of carbon nanotubes can degrade the thruster characteristics. On the other hand, carbon nanotubes are very attractive due to the properties of the carbon material, and that material should obviously undergo active testing in EP devices, taking into account the above-mentioned encouraging results on wear resistance.

Thus, the dense brushes of vertically aligned nanotubes demonstrate quite attractive and fascinating properties when tested for wear resistance under the action of energetic ion flux in the Hall thruster channel. Notably, the carbon nanotubes are conductive and can change the acceleration mechanism (the wall conductivity is not an inadmissible condition, but it significantly affects the process^70)^. Undoubtedly, the first encouraging experiments^67,69^ force further complex investigations to cast light on the application of carbon nanotube brushes and other graphene-containing materials in EP engineering.

Graphene nanowalls is one more material potentially capable of enhancing channel wear resistance. Along with the carbon nanotubes, graphene and graphene flakes may be attractive for enhancing the channel wall wear resistance. Graphene is the strongest material in nature and carbon and carbon nanotubes are particularly resistant to ion sputtering; this makes the surface-grown graphene flakes (nanowalls) extremely attractive candidates for wear-resistance testing in the Hall thruster channel. Dense patterns of carbon nanowalls can be formed directly on the ceramic surface, with or without metal catalyst particles^71^ if required. Direct growth on ceramic and metallic^72^ materials is also possible^73^. The exemplary graphene patterns grown on alumina are shown in Fig. 2, left panel. They consist of nearly vertical graphene flakes attached by one edge to the face surface of the ceramics, with the other edges being open. Graphene nanowall patterns have not yet been subjected to extensive ion flux and wear testing in real EP devices, but positive results of the experiments with other carbon-containing, and especially graphene-like and diamond-containing nanostructures, encourage further work in this direction. Similar to other ultramodern techniques, graphene and nanotubes should find their deserved place in EP technology.

Table 1 shows a list of the presently used and tested materials and nanomaterials for application as a coating on the acceleration channel. Among then, the newly synthesized ultra-BN (uBN) material fabricated by plasma-assisted CVD (chemical vapor deposition) process demonstrates excellent wear and discharge efficiency characteristics. With extremely low surface roughness and very low erosion coefficient (the total wear rate of several nm × h^−1^, order of magnitude lower than that of BN), the ultra-disperse uBN appears to be the best candidate for the highly efficient, long-life thrusters. The operational tests of this material are in progress, and a flight test is planned.

Apart from the material-related approaches, a so-called magnetic shielding technique was recently demonstrated^74^. Sophisticated selection of the shape of the magnetic field at the exit of the accelerating channel, and the proper profiling of the channel exit ensure a significant decrease in the intensity of ion bombardment to the channel walls, and hence, substantial decrease in the wear of channel walls. As a result, the service life of the channel can be essentially increased, thus significantly prolonging the thruster lifespan without notable drop in the performance characteristics. Schematics of the magnetic shielding technique is depicted in Fig. 2, right panel. Importantly, magnetic shielding allows to operate the thruster at higher voltage levels, i.e., the specific impulse that is in general proportional to the discharge voltage could be increased. Moreover, magnetic shielding partially precludes electrons from contacting with the walls and hence, allows changing the wall material (to metals or carbonaceous materials, or cheaper ceramics) without reducing the thruster characteristics. On the other hand, this technique requires more complex magnetic topology, which could make the EP system somewhat more complicated and potentially less reliable. However, potential significant benefits of this technique call for further studies since the high-voltage operation requires new resistant materials even in the magnetic shield mode. Further, both new materials and sophisticated magnetic field topology are necessary to achieve the dual-mode operation described in more detail below^75^.

### Longer life and higher cathode current via advanced materials

Cathode is the second electrode used in any type of static thruster to apply negative potential to the discharge zone and in some cases, to supply the flux of electrons to the discharge zone where they are magnetized, as well as to compensate the space charge of (non-magnetized) ions; that is why the cathodes on Hall thrusters are sometimes called neutralizers. As a rule, a couple of cathodes (a working one and a spare one) are usually installed outside of the main body of the thruster, as shown in Box 2. Cathodes may also be installed well outside the thruster (to ensure better thruster characteristics) and thus will require strong protection^76^. Optimization of the cathode position is required to ensure the highest thruster performance characteristics^77^.

**Fig. 3:**
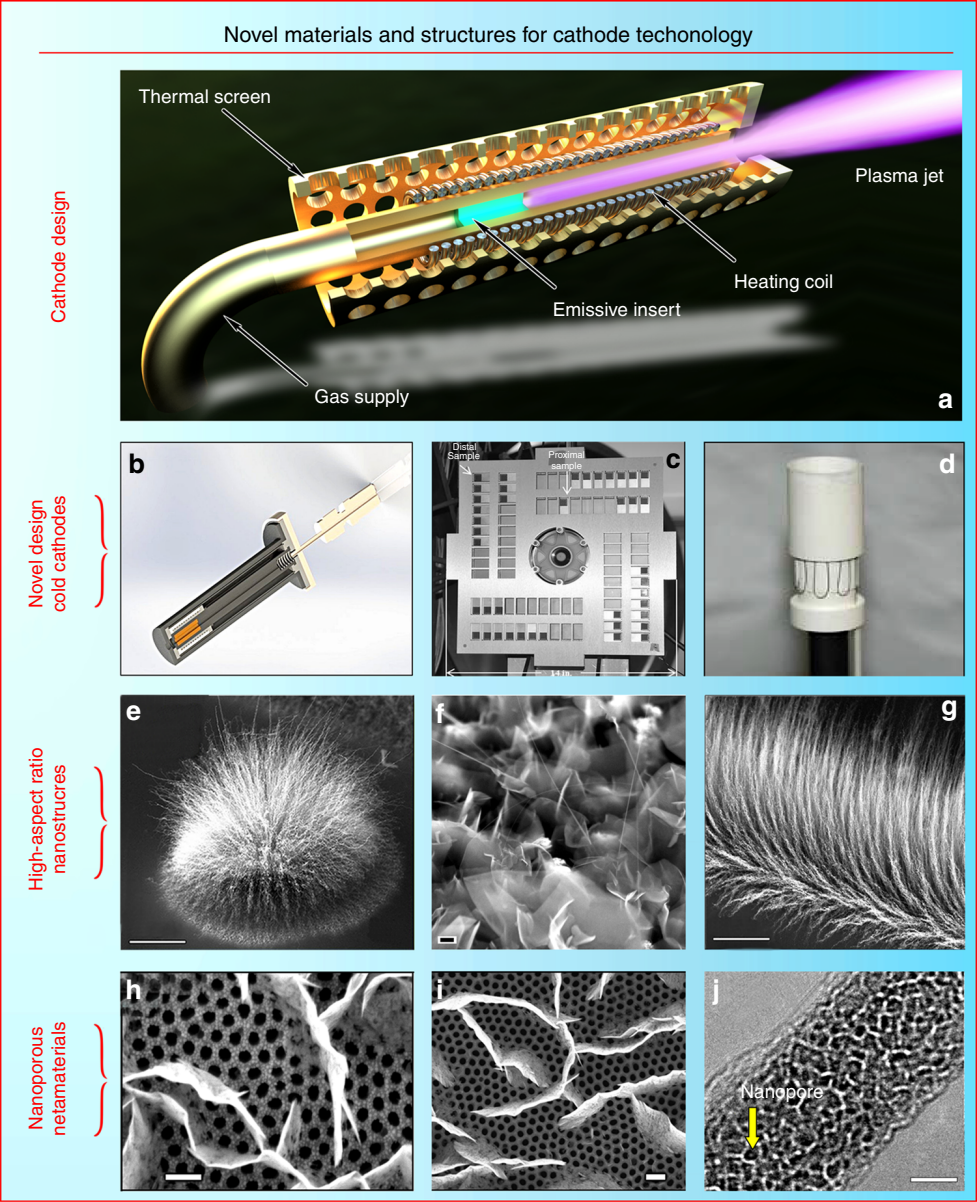
Novel materials for cathode technology. **a** Schematic of the commonly used thermosemissive cathode. The typical electron emission cathodes incorporate a central tube (1–5 mm dia.) with a porous highly emissive insert (typically lanthanum hexaboride LaB6) and a heating coil installed around the tube. Gas (with the flux rate of several percentage points of the total thruster propellant consumption) is supplied into the central tube, which is heated by the coil. The emissive insert ensures thermoionization of the gas exiting the cathode orifice. When the thruster is working (discharge is sustained in the chamber), plasma forms a narrow jet connecting the cathode orifice with the main discharge zone, and electrons pass to the discharge via the plasma jet. The cathode includes a thermal screen to reduce the heat loss to space due to ionization. In addition, it incorporates elements and parts to attach the cathode to the thruster and the gas feed system. **b** New highly efficient cathode with a cold nanoporous metal emissive element (under testing at the PSAC/SPCS Lab). **c** Carbon nanotube cold cathodes in a Hall effect thruster environment. Reproduced from ref. ^135^. (Copyright 2013, ERPS). **d** Classical cathode with a heating coil and an emissive La-B insert. Reproduced from ref. ^136^. (Copyright 2011, ERPS). **e** Cluster of carbon nanotubes serving as a cold emitter. Scale bar, 50 µm. Reproduced from ref. ^90^. (Copyright 2012, Elsevier). **f** Nanocrystalline graphite in an advanced field-emission spacecraft cathode. Scale bar, 200 nm. Reproduced from ref. ^137^. (Copyright 2011, ERPS). **g** Long nanotubes on silicon. Scale bar, 15 µm. Reproduced from ref. ^90^. (Copyright 2012, Elsevier). **h**, **i** Vertically aligned graphene flakes grown directly on the nanoporous alumina. Scale bars, 200 nm. Reproduced from ref. ^129^. (Copyright 2014, Elsevier). **j** Single-crystalline MoO_3_ nanowire. Scale bar, 10 nm. Reproduced from ref. ^93^. (Copyright 2012, RSC)

The cathode in the present-day thruster (see the schematic of the commonly used thermosemissive cathode in Fig. 3a) is a very power- and current-loaded device, which should provide reliable service for very long periods of time. One of the typical cathodes with the LaB_6_ electron emitting insert (one of the most common materials used in the emissive cathodes) was designed to ensure the lifetime of 100,000 h at the discharge current of 40 A, thus can be used to power a 12.5-kW Hall thruster under development for the proposed Asteroid Redirect Robotic Mission^78^. The cathode can be used as a low thrust thruster itself^79^. Cathode erosion also represents a problem reducing the service life^80^.

Thus, the key challenges for the cathode (and consequently possible directions for the enhancement of thruster efficiency via upgrading the cathode unit) consist in the reduction or total elimination of propellant consumption via the cathode, significant boost of the cathode service life, and the reduction of heat loss from the incandescent parts.

Can nano help here? Let us examine how nanomaterials and nanotechnology can help achieve these goals.

Reduced gas consumption may be ensured through the use of nanoporous materials, nanotubes, and graphene. The best solution for this problem is the total elimination of gas flux through the cathode by using high-emissive materials and surface structures. Numerous tests have demonstrated that usual solid and microporous materials cannot ensure notable enhancement of characteristics compared with the commonly used LaB_6_ material. However, encouraging experimental results have been obtained by testing various nanostructured and surface-engineered materials, such as nanoporous metal emissive elements (Fig. 3b). Carbon nanotubes were also successfully tested as field-emission electron sources operating without or at reduced gas supply^81^ (Fig. 3c shows the photograph of carbon nanotube cold cathode installed on a Hall effect thruster). Moreover, multiwall carbon nanotube emitters were tested directly for the use in spacecraft cathode units;^82^ the tests were conducted specifically for the operation in the Hall thruster plume environment^83^. Other nano-engineered materials also demonstrated promising electron emission properties, e.g., ultrananocrystalline diamonds^84^ and nanocrystalline diamond-coated silicon tip arrays^85^. Nano- and micro-engineered materials relevant to EP were also tested for their electron emission capabilities^86^. Vertically aligned graphene^87^ has also demonstrated inspiring results in electron emission tests^88^. Investigations related to the reliability and failure mechanism of the carbon nanotube-based cathodes are also undergoing active exploration^89^. Therefore, carbon nanotubes (both single- and multi-walled) and vertically aligned graphene flakes are good candidates for significantly enhancing thruster efficiency due to the propellant-free regime (we recall here that the propellant consumption by the conventional incandescent cathode can reach 10% for small thrusters, and usually accounts for 3–5% of propellant consumed for larger devices). In spite of many successful experiments referenced here, more efforts are required to utilize the full potential of vertically aligned nanostructures in cathode-related applications, and newly developed nanostructures and patterns can be much more efficient than those already tested classical cathodes with a heating coil and an emissive La-B insert (Fig. 3d).

An example of a novel and potentially efficient nanomaterial for cathodes is the cluster-grown carbon nanotubes and nanocrystalline graphites. These clusters could be arranged into various shapes by a simple mechanical drawing (using e.g., mechanical tools or a laser beam) of the required configuration on the surface. Figure 3e, f, g show the SEM (scanning electron microscopy) images of nanotube and nanocrystalline graphite clusters. Such aggregates are extremely promising for emission-related applications. Scanning and transmission electron microscopy characterizations show that the longest nanotubes reach several hundred microns in length, the array density reaches 1000 nanotubes per l μm^2^, and diameters of the nanotubes were in the range of 15–50 nm, with up to 20 walls. Importantly, dense linear brushes of nanotubes could be produced over the entire sample size of 10 mm. More detail about the growth process can be found elsewhere^90^. Further studies of these and other nanotube and graphene patterns and surface structures are needed to make a definitive conversion from contemporary propellant-consuming cathodes to novel, cold, propellant-free nanomaterial-based cathodes. More examples and a detailed description can be found in Fig. 3.

When the propellant-free design is inapplicable, complex nanostructure-based surface systems (metamaterials) may be proposed for application in thruster cathodes, with one of the example systems shown in Fig. 3h, i. A dense pattern of the vertically aligned graphene flakes was grown on the nanoporous^91^ alumina membrane. Propellant can be supplied directly through the nanoporous alumina, and electrons will be emitted from the acute edges of the graphene, which are emission-capable structures as was demonstrated in direct experiments^88^.

Longer life of cathode could be also reached by the use of ultra-nanoporous inserts. Emissive inserts work when electrons exit an emissive material from the surface; hence, larger surface area per volume unit of the insert could be beneficial due to the lower required heated volume and more efficient electron emission. Here, we demonstrate an example of ultra-nanoporous highly-crystalline nanowires with uniformly distributed nanopores in the 3 nm range produced by electron beam exposure (Fig. 3j). In the example, single-crystalline MoO_3_ nanowires were synthesized by a simple, environmentally friendly plasmoxy-nanotech process^92^ by direct exposure of a pure Mo foil to reactive oxygen plasmas in a Pyrex glass reactor. After synthesizing the single-crystalline MoO_3_ nanowires, they were transferred to the transmission electron microscopy grid for the electron beam irradiation. This method is based on the electron beam-driven oxide-to-sub-oxide and then sub-oxide-to-metallic transition that can be controlled by the electron beam exposure. The diameters of the resulting pores range from 2 to 5 nm, with a Gaussian-like distribution and an average size of around 3.6 nm. More details on the process and performed characterizations can be found elsewhere^93^. This nanomaterial brings together several beneficial properties, namely a unique ultra-nanoporous structure (nanopore size of 2–5 nm) and an extremely high porosity coefficient reaching 0.75. This technique can potentially be used to produce other materials, including those suitable for the emissive inserts, and very efficient inserts may be fabricated.

**Fig. 4:**
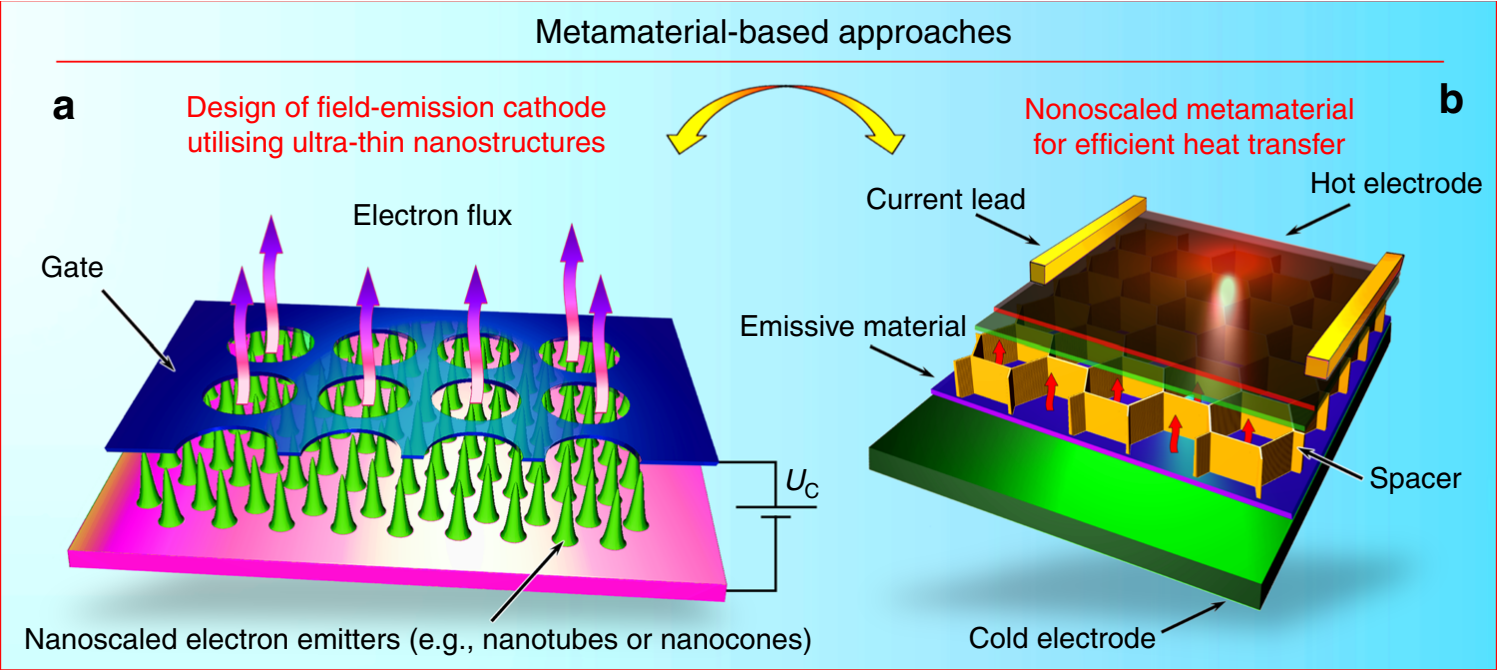
Metamaterial-based approaches. **a** Design of a metamaterial-inspired cold electron emitter incorporating the dense array of emitting elements (e.g., carbon nanotubes or metal-tipped nanocones) grown directly on the electrode, and the gate electrode with µ-sized orifices. Due to strong electric field enhancement on the long nanosized structures, significant current densities could be obtained without external heating. **b** Design of the nanoscaled metamaterial capable of reversal heat transfer by electron emission. This structure may include a nanoporous membrane (a porous insert) capable of containing some amount of a highly emissive material usually working in a liquid state (e.g., cesium). The nanoporous membrane with the emissive material in pores is placed directly onto the solid cathode, and the spacer separates this membrane and the anode with the low-emissive material. The low-emissive material (e.g., tungsten) covers the anode surface. The cathode (the cold electrode in green) has a temperature lower than that of the anode (the hot electrode in red). Electron current emitted from the cathode flows through the inter-electrode gap to the anode. It is evident that such metamaterials could create strong heat barriers in the systems where heating over some limit (e.g. Curie temperature) should be avoided. Reproduced from ref. ^138^. (Copyright 2016, Wiley)

Nanoscaled metamaterial could help to reduce the heat losses. The incandescent cathode wastes a great deal of heat energy through radiation from the cathode’s hot parts into space. However, ideally, the entire heat generated in the cathode should be used within (at the emissive insert) and should not be released from the outer parts of the unit. Any type of currently used heat protections essentially provides passive protection. Using nanoscaled metamaterials, active heat protection physically based on heat pump principles can be designed. Specifically, external energy should be spent to transfer the heat from colder parts to heated parts by, e.g., nanoscaled high-temperature metamaterial capable of reversal heat transmission^94^. During the metamaterial operation, an electrical potential dependent on the distance between surfaces and the current density is applied between the anode and the cathode to sustain the current in the gap. As a result, heat may be transferred from the hot surface directly to the colder part by the electron current in the gap, i.e., this metamaterial works as an electronic heat pump. As a result, heat that leaks from the hot cathode to space can be significantly reduced. We stress that this is only a concept under active investigation^95^. Further investigations will be needed to design, test, and implement the novel heat-transferring systems in the material form factor. A schematic of the metamaterial proposed for the active heat pumps is shown in Fig. 4.

### Adaptive and self-healing thruster via nanomaterials

As previously mentioned, the nature of the wall material can be used to classify Hall-type thrusters as TAL featuring metallic channel walls, and thrusters with ceramic walls (stationary plasma thruster, SPT). These two types exhibit slightly different characteristics and require somewhat different design approaches, and as such, these devices could occupy specific application niches and be successfully used in space exploration for various missions. In general, the total difference in performance between ceramic-walled SPT-type thruster and metal TAL is not so significant. Nevertheless, both types are attracting attention, and among other advantages, TAL can ensure efficient operation at higher (up to several kV) voltage, so the specific pulse (exhaust velocity) will be higher. In turn, this may be advantageous for the missions requiring elevated specific impulse at somewhat lower thrust, such as orbit keeping or debris removal^96^ for several years at limited onboard power and limited mass. At present, the TAL-type thrusters are underexplored, yet numerous studies indeed demonstrate their better operability at significantly increased voltages^97,98^. Even within the same thruster type, the wall material characteristics may influence thrust, exhaust velocity, and thruster efficiency^99,100^.

Evidently, TAL is not congruent to a standard SPT thruster with conducting walls, and apart from the wall material state, some other adjustments (e.g., shift of the anode to shorten the discharge zone to the TAL-optimized configuration, reshaping the magnetic field by switching coil sections, and so on) will be required to ensure efficient TAL operation in the proper mode, i.e., in the anode layer regime; nevertheless, such transformations appear to be quite attainable in flight^101^.

Is it possible to actively control processes in thrusters by nanomaterial-based techniques? Evidently, many of the present-day nanomaterials and surface-engineered systems could be tested for the active control of the thruster operation. Acceleration channels made of various materials such as solid BN, diamond- or ultrananocrystalline diamond-coated walls, or carbon nanotubes/graphene-protected surfaces can exhibit notably different surface morphologies, roughness, and electrical conductivity, and importantly, reasonably distinct electron emission features also determined by the presence of acute tips (e.g., on carbon nanotubes), sharp edges (e.g., on graphene flakes), and sharp facets (e.g., on nanodiamond crystals). Undoubtedly, control of such a set of unique properties can lead to thrusters with a wide operational range, and moreover, could allow adaptive switching between the operational modes.

Importantly, most of the nanostructures and nanomaterials mentioned above are routinely fabricated, or could be fabricated using low-temperature plasmas with properties that are quite similar to plasma properties within operating Hall thrusters, specifically: plasma density of ≈10^18^–10^19^ m^−3^, electron energy up to 10–20 eV, and process voltage of about several hundreds of volts. Granted that different feedstock gases must be used to accomplish propulsion or nanosynthesis, we still find the similarities quite encouraging. Indeed, Hall thrusters have already been successfully tested for nanofabrication, and hence, desired nanostructures and nanomaterials could be in principle synthesized directly in the discharge (acceleration) channel.

Thus, we propose the concept of the so-called adaptive thruster, i.e., the thruster that is capable of changing the main discharge type (thruster type) by actively adapting the channel wall parameters by synthesizing required nanomaterials in its own discharge, then depositing them onto specific locations inside the channel, and finally modifying the channel wall surface characteristics by direct interactions with its own channel plasma. In more detail, the electric, magnetic, and gas supply systems of the thruster should be designed and tuned to be capable of ensuring temporary transition of the discharge from acceleration to nanosynthesis mode (changing the gas composition by adding the nanomaterial precursor such as, e.g., methane and hydrogen for carbon nanotube, graphene, or diamond synthesis, and preprograming synthesis protocol) to ensure optimum nanomaterial nucleation and growth conditions, and restoring discharge to the acceleration mode after adjusting the channel wall to the new required thrust/velocity conditions.

Graphane can be a key material to realize this task. The ability of switching between TAL and SPT operational modes is a possible implementation of an adaptive thruster concept. As the wall material (a ceramic or a metal) is the major discriminant of these modes, the thruster ability of adapting the conductivity of its own wall material would enable the realization of adaptive features. For instance, hydrogenation of the surfaces of graphene flakes on the channel walls by the thruster discharge plasma may ensure quick and reversible transition from insulating to conductive states and vice versa, thus would provide the ability to switch between TAL and SPT regimes directly in flight and possibly, without interruption of the thruster operation. Indeed, it was already demonstrated that graphene can react with atomic hydrogen, and this reaction transforms graphene (which is a perfect electrical conductor) into graphane, an insulator made out of a two-dimensional compound of carbon and hydrogen (i.e., hydrogenated graphene)^102^. This reaction is also reversible, and the graphene structure is maintained when graphane is formed by attaching hydrogen atoms to graphene. Importantly, this reaction requires low-temperature plasma similar to that present in the Hall thruster discharge. In the original experiment^103^, 10% admixture of hydrogen in argon was used to convert graphene into graphane. Thus, by changing the plasma parameters in the thruster discharge, it is possible to change the conductor/insulator state of the acceleration channel surface by depositing graphene flakes. Xenon, which is commonly used in EP is similar to argon (both are inert gases with close ionization potentials and both sustain the discharge in crossed E × B fields at similar voltages), could be considered as suitable for a similar hydrogenation process. The hydrogen admixture in the gas of an operating thruster could be short, since only surface hydrogenation is required. As hydrogen source, metal hydrides (i.e., MgH_2_) can be used. At present, there is no scientific evidence regarding graphane behavior under the effect of the energetic ion flux, but encouraging results show that graphane is relatively stable (as it requires annealing for at least 24 h at 450 °C)^103^. Both vertically aligned graphene flakes, as well as graphene-like inlaying films can be proposed for adaptive thruster applications. In any case, the use of such materials should be explored due to the potential importance of the proposed in-operation and in-flight conversion of TAL to SPT.

Alternatively, an insulating film could be temporarily deposited onto the metal walls to transform a TAL-type device into a SPT-type. For this purpose, a small amount of silane (silicon and hydrogen containing gas, SiH_4_) and oxygen (or e.g., water vapor) can be premixed with the inert propellant (xenon). Indeed, it was already experimentally demonstrated that addition of silane (10 p.p.m. only) in the low-temperature plasma results in the efficient nucleation of silicon nanocrystals with the characteristic size of several nanometers^104^. Figure in Box 4 shows the experimental setup, as well as a photograph of the discharge and a scanning electron microscopy image of the nucleated silicon nanoparticles (mean diameter <5 nm). By tailoring the plasma properties, different nanoparticle characteristics, such as chemical composition and crystal structure, can be achieved. Oxidation can then be easily achieved with a very small amount of oxygen or even water vapor^105^.

The application of a thin metal film onto the channel wall surfaces from the discharge plasma is another approach to adaptive thruster systems. Indeed, this approach will require admixture of a metal-containing gas^106^ or evaporated metal-containing liquids^107^ capable of producing suitable metal precursors in the discharge. A metal film deposited onto the wall surface will ensure the transition from insulating to conductive states, but the wear resistance of pure metal is low and thus could be used for short-term regime switching.

Deposition of such oxidized nanoparticles onto the metallic walls of the acceleration channel will create electrically insulating layer and thus transform the TAL device into SPT. Notably, Hall thrusters with a significant addition of silica on the channel walls is quite functional, albeit with somewhat lower efficiency.

Is it possible in a real thrusters? Yes, provided that an efficient control over the processes in plasma-wall sheath is ensured, e.g., by the magnetic shielding that was encouragingly demonstrated for the thruster channel. Proper control of the plasma configuration^108^ could make it possible to temporarily separate the main discharge zone from the nanoparticle nucleation and deposition area, thus ensuring direct growth of the nanostructures in the discharge and the following deposition onto walls. Extended experimental and theoretical studies will be required to bring these concepts to life.

Using similar design solutions, it is possible to fabricate electrodes with, for instance, thin layers of carbon mixed with layers of traditional BN. Such sophisticated structures enable active control over the near-wall conductivity^109^, and hence, enhance the discharge and ion acceleration. Furthermore, the replacement of thin carbon layers with the graphene (and possibly, multi-walled graphene flakes^110^ could potentially enhance the characteristics due to higher mechanical strength, as well as electrical^111^ and heat conductivities, intrinsic to graphene^112^).

### Box 4: successful conceptual experiments toward self-healing

**What is self-healing?**

Self-healing is the ability of a material, part, or system to restore its original integrity and functionality after damage without any direct external effect or in response to a small change in the operating regime that does not affect the working capacity of the entire system. Self-healing process may occur uninterruptedly during normal operation of the system, or periodically during specially organized sessions when the operation cyclogram permits.

**Was it already demonstrated in conditions similar to those found in plasma thrusters?**

Yes, it has already been successfully demonstrated! We have designed and conducted a series of dedicated conceptual experiments to demonstrate that the discharge system can restore its functionality during the normal operation. Specifically, we have tested the behavior of a micro-discharge system (see schematics above) consisting of electrodes forming a discharge gap of 1–2 mm on the surface of silicon or BN ceramics that is typical for EP applications. A thin carbon layer (about 0.1 μm) was applied between the electrodes to ensure initial breakdown. After application of the first electric pulse (typically, 100 V), the breakdown occurs and the carbon layer is completely removed from the gap; nevertheless, a new thin layer of tungsten restores the conductive state between electrodes, thus preparing the system for the new pulse. Similar systems often use a special consumable electrode or even more complex device to maintain the readiness to produce discharge (and hence, to supply the pulse of thrust); in our case, a primary function of the system was maintained by the system’s behavior, thus demonstrating self-healing at the level of the material. Adapted with permission from Teel et al^115^. Copyright 2017, AIP.

**Can plasma nucleate nano- and µ-particles directly in the discharge?** 
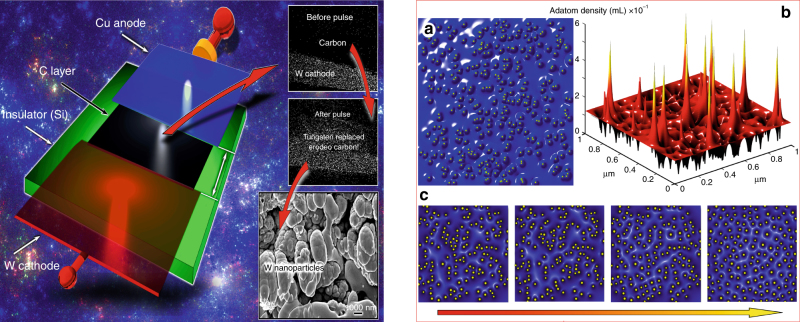


Yes, this was demonstrated in the experiments**—**SEM image below sows the carbon nanoparticles synthesized in plasma and deposited to plasma-exposed surfaces (adapted from Hundt et al^145^). Direct synthesis and deposition of complex nanostructures such as carbon nanotubes and graphene nanoflakes is also possible in plasmas similar to that present in Hall thrusters^146^. In a typical experiment, graphene flakes were grown in a plasma with electron energy of several eVs and electron densities of about 10^17^ m^−3^, i.e., very close to the thruster plasma parameters. Moreover, the morphology of the graphene patterns on the surface can be efficiently controlled by the applied electric field, as it was already demonstrated in the experiments where the flakes were aligned by the electric field^147^. Nevertheless, this is a very complex task that requires sophisticated control of the plasma parameters and plasma fluxes in the channel.

**What is self-organization?**

Self-organization is a process that leads elements to order and, eventually, it results in the establishment of a system of a higher order. In simpler words, self-organization is the process of origination of larger, more complex system out of smaller and simpler parts, due to the internal driving forces. Formation of a thin layer from small particles is an example we are interested in.

**What drives self-organization on surface?**

A pattern of material fluxes on surfaces is the main reason for self-organizing behavior in an array of nanoparticles. Since these fluxes are mainly governed by the adatom density and electric field patterns near the surface, the surface diffusion and electric field may be considered as the main driving forces for the self-organization. To control the self-organization in nanoarrays, proper adatom/adradical density and electric field patterns should be created by sophisticated tailoring of the process parameters, mainly the plasma-surface interaction.

**How to control self-organization on surfaces?**

Calculated distribution of the electric field in the simulated pattern of nanoscaled irregularities on a plasma-exposed surface, and (**b**) three-dimensional visualization of the nanoscaled irregularity pattern and adatom density profile on the surface. Pattern size is 1×1 μm. Strong peaks of the near-surface electric field reaching 10^8^ V × m^−1^ (**a**) and adatom density (**b**) causes rapid, intense material redistribution resulting in healing of wear traces by filling them with new material delivered directly from plasma and diffused across the hot surface. Levchenko et al^148^. Copyright 2013, American Chemical Society.

**Do complex nanostructures demonstrate self-healing behavior?**

Yes, self-healing of BN nanotubes was directly observed.

Boron nitride nanotubes have structure similar to that of well-known extra-strong carbon nanotubes and thus feature similar properties (Golberg et al^149^). High-resolution TEM (transmission electron microscopy) images below show self-healing of kinked BN nanotube. The nanotube fractured by the action of an external force undergoes self-healing, with no trace of kink found at the affected area (Golberg et al^150^). With very high tensile strength similar to that of carbon nanotubes, and excellent electrical characteristics ensuring efficient plasma acceleration in Hall thrusters, BN nanotube can find many applications in the future thrusters.

### Perspective

The NASA’s 2015 Nanotechnology Roadmap includes, among other important concepts, a new paradigm of paramount importance, a so-called concept of a self-healing spaceship. This covers several mainlines, in particular self-cleaning surfaces, self-healing repair mechanisms, and self-repairing surfaces and materials. Initial research efforts by NASA and associated research institutions have demonstrated self-healing materials that were capable of repairing the punctures in several seconds^113^. Moreover, other possible self-healing approaches for EP systems were explored; at the thruster level, self-healing field-emission neutralizers (cathodes) for EP devices were developed by NASA in collaboration with Aerophysics, Inc. in the framework of the NASA Small Business Innovation Research (SBIR) contracts^114^.

The implementation of self-healing and self-restoring materials and systems to the entire spacecraft makes the self-healing approaches compulsory for subsystems such as thrusters that are the critical elements of the propulsion system. Furthermore, just the wear-affected acceleration channel walls that actually limit the thruster life are the ultimate elements to be examined in terms of incorporation of self-healing materials and techniques. Apparently, we are currently at the stage of problem formulation and determining the general angle-of-attack to ensure the development of the most general approaches; nevertheless, reasonable efforts are to be applied now in the framework of the NASA’s self-healing spaceship concept.

Specifically, we are proposing the self-healing approach (plasma-enabled healing) similar to that suggested for modification of the acceleration channel material and in-flight switching mode of thruster operation between the TAL and SPT regimes; specifically, self-healing should be enabled by plasma nucleation and deposition of various nanostructures and nanomaterials. The difference is that in the case of self-healing, the deposition of plasma-nucleated nanomaterials (most probably, as small as possible) or appropriate ions (e.g., silicon oxide formed in plasma with silane added) should be ensured at proper places (in wear damage locations only), and moreover, certain surface processes should be activated and sophisticatedly controlled to conduct efficient wall healing, i.e., surface repair by the controlled re-deposition of the nanomaterial. Importantly, the first experiments have demonstrated this mode in the thruster-like conditions^115^.

Two different regimes of the thruster self-healing operation may be considered, depending on the specific wall material, propellant, thruster type, and plasma parameters, namely the concurrent mode self-healing conducted continuously during the thruster work (the worn areas are restored immediately after reaching some detection threshold), and the sessional mode based on healing/restoration sessions conducted periodically, when the wall wear reaches the critical point affecting thruster’s operational characteristics.

In any of the above modes, several key processes should be activated and controlled, such as nucleation of the appropriate nanoscaled particles (most probable for the sessional self-healing) or appropriate ions (for concurrent self-healing); delivery of thus-formed particles to the most appropriate locations (i.e., to the most worn spots on the wall surfaces), this is important for both concurrent and sessional modes; and surface processes such as material diffusion, coalescence, and incorporation in the elements of wall surface (worn pits), ensuring efficient repair of the plasma-affected surfaces. Thus, the general route could be formulated as nucleation → delivery → repair.

Detailed examination of all these processes is obviously outside the scope of our work, the aim of which is to set the general directions, suggest potential approaches, and stimulate discussion within this highly promising area of research. Nevertheless, we would like to take this opportunity to point out and briefly characterize the physical mechanisms at the heart of the above process. We should highlight that surface self-healing is essentially a manifestation of self-organization^116^ and self-assembly;^117^ moreover, self-healing is a surface-based self-organizational process^118^ and plasma exposure can effectively produce strong driving forces^119^ to drive self-organization on surfaces^120^.

Nucleation of the appropriate nanoscaled particles was in short discussed in the above subsections, and it was shown that the nucleation of various nanostructures and nanocrystals is possible in the plasma environment similar to that of the present-day Hall thrusters^104^.

The delivery of plasma-nucleated charged nanostructures to the most appropriate locations, as well as surface processes such as diffusion and material incorporation, could be achieved via non-uniform electric fields that develop at the plasma-solid interface and in particular enhanced by rough surface. It was already demonstrated that electric field could control movement of ions and nanoparticles near surfaces, and eventually ensure material deposition onto preferred locations within the nano-textured surface pattern^121^. Detailed numerical simulations of the electrical field patterns (Figure in Box 4) and irregular fields of adsorbed atoms (adatoms) on the plasma-exposed rough surfaces were verified by experimental studies on the plasma-driven surface restructuring and confirmed intense self-organization processes^122^ under actions of low-temperature plasma^35^ and surface electric potential^123^.

Briefly, strong peaks of the near-surface electric field and adatom density causes rapid, intense material redistribution resulting in healing of worn traces by filling them with the repairing material. Importantly, the efficient incorporation of the repairing material into the worn traces requires plasma and electron irradiation for degasing of the surface and for the creation of dangling bonds^124^. In general, surface-based processes are relatively well described and numerous studies were conducted to show how the plasma composition, surface temperature, and other parameters influence surface restructuring. Ultimately, special arrangements should be designed to fully control the entire process, e.g., heaters for ensuring the optimum temperature and additional aids to adjust the magnetic field during the self-healing session.

Apparently, significant stumbling blocks and constraints should be expected during implementation of adaptive and self-healing thruster strategies. Indeed, the adaptive and self-healing strategies for boosting mission efficiency and significant extension of the thruster lifetime would clearly complicate the entire system and hence, disadvantages should be carefully considered and taken into account. Moreover, not every thruster system could be suitable for such an upgrade, and it is quite possible that the drawbacks related to the increase in weight and complexity, and consequent lower weight efficiency and lower reliability could make some systems not appropriate for the adaptive and self-healing technologies.

In general, the alternative approach could include redundancy (installation of additional thrusters to prolong the life of the entire system and change the operation mode by switching the thrusters), which is commonly used in similar complex technical systems. Evidently, a detailed systematic analysis will be required in each specific case to determine the applicability and practicability of the adaptability and self-healing strategies, based on a spectrum of parameters. Indeed, compromise between mass increase due to additional thrusters, connections, frames, and so on, in the case of the redundancy approach, and mass increase due to additional gases, gas tanks, valves, power supply units, control systems, and so on, will be required to maintain adaptability and self-healing processes. Moreover, compromise between life increase due to self-healing and life decrease due to lower reliability of the whole system after introduction of additional self-healing subsystems should also be considered.

Detailed systematic analysis should be performed in each specific case and specific mission, and possible stumbling blocks and constraints should be detected, analyzed, and assessed to consider the applicability of the adaptability and self-healing techniques and their benefit against the increased complexity. Indeed, the self-healing and self-adjusting space system will represent a next level of complexity, in fact being in part a biomimetic system.

Moreover, the study of the critical processes within an adaptive and self-healing space thruster system presents a considerable experimental challenge, with significantly more effort required to first demonstrate some self-healing functions at the system level. However, given the potential benefits, this is a worthwhile effort since it could potentially revolutionize the entire approach to designing and building the spacecraft systems and other space-based platforms. Furthermore, while creation of the flight-ready self-healing thruster is a scientific and engineering challenge of immense complexity, the state-of-the-art techniques make it conceptually possible.
