# Publisher Correction: Recent progress and perspectives of space electric propulsion systems based on smart nanomaterials

**DOI:** 10.1038/s41467-018-03818-4

**Published:** 2018-04-10

**Authors:** I. Levchenko, S. Xu, G. Teel, D. Mariotti, M. L. R. Walker, M. Keidar

**Affiliations:** 1 Plasma Sources and Applications Centre, National Institute of Education, Nanyang Technological University, 1 Nanyang Walk, Singapore, 637616 Singapore; 2 School of Chemistry, Physics, and Mechanical Engineering, Queensland University of Technology (QUT), 4000 Brisbane, QLD Australia; 3 Department of Mechanical and Aerospace Engineering, The George Washington University, Washington, DC 20052 USA; 4 Nanotechnology and Integrated Bio-Engineering Centre (NIBEC), Ulster University, BT37 0QB Newtownabbey, UK; 5 School of Aerospace Engineering, Georgia Institute of Technology, Atlanta, GA USA

Correction to: *Nature Communications* 10.1038/s41467-017-02269-7; published online 28 February 2018

The original PDF version of this Article had an incorrect volume number of ‘8’; it should have been ‘9’. This has been corrected in the PDF version of the Article. The HTML version was correct from the time of publication.

